# Complex Analyses of Short Inverted Repeats in All Sequenced Chloroplast DNAs

**DOI:** 10.1155/2018/1097018

**Published:** 2018-07-24

**Authors:** Václav Brázda, Jiří Lýsek, Martin Bartas, Miroslav Fojta

**Affiliations:** ^1^The Czech Academy of Sciences, Institute of Biophysics, Královopolská 135, 612 65 Brno, Czech Republic; ^2^Department of Informatics, Mendel University in Brno, Zemědělská 1, 613 00 Brno, Czech Republic; ^3^Department of Biology and Ecology/Institute of Environmental Technologies, Faculty of Science, University of Ostrava, Ostrava, Czech Republic

## Abstract

Chloroplasts are key organelles in the management of oxygen in algae and plants and are therefore crucial for all living beings that consume oxygen. Chloroplasts typically contain a circular DNA molecule with nucleus-independent replication and heredity. Using “palindrome analyser” we performed complete analyses of short inverted repeats (S-IRs) in all chloroplast DNAs (cpDNAs) available from the NCBI genome database. Our results provide basic parameters of cpDNAs including comparative information on localization, frequency, and differences in S-IR presence. In a total of 2,565 cpDNA sequences available, the average frequency of S-IRs in cpDNA genomes is 45 S-IRs/per kbp, significantly higher than that found in mitochondrial DNA sequences. The frequency of S-IRs in cpDNAs generally decreased with S-IR length, but not for S-IRs 15, 22, 24, or 27 bp long, which are significantly more abundant than S-IRs with other lengths. These results point to the importance of specific S-IRs in cpDNA genomes. Moreover, comparison by Levenshtein distance of S-IR similarities showed that a limited number of S-IR sequences are shared in the majority of cpDNAs. S-IRs are not located randomly in cpDNAs, but are length-dependently enriched in specific locations, including the repeat region, stem, introns, and tRNA regions. The highest enrichment was found for 12 bp and longer S-IRs in the stem-loop region followed by 12 bp and longer S-IRs located before the repeat region. On the other hand, S-IRs are relatively rare in rRNA sequences and around introns. These data show nonrandom and conserved arrangements of S-IRs in chloroplast genomes.

## 1. Introduction

Inverted repeat sequences (IRs) play an important regulation role in genomic DNA [1]. It has been demonstrated that short IRs (S-IRs) are essential for DNA replication in both prokaryotes and eukaryotes [2]. Depending on their length, sequence, and crowding conditions, S-IRs can form a local DNA structure called cruciform, which is a target of numerous DNA-binding proteins with important regulatory functions [3]. Among these proteins are cruciform resolving proteins like* E. coli* enzyme RuvA [4, 5] and proteins important for human diseases including the 14-3-3 [6] and p53 protein families [7, 8]. Similarly, high mobility group- (HMG-) like cruciform binding proteins have also been purified from plants [9] and it was demonstrated that IRs in chloroplast DNA (cpDNA) genomes play a role in evolution and various regulatory processes [10, 11]. Both chloroplasts and mitochondria retain their own genomes and transcription machinery. S-IRs in mitochondrial DNA (mtDNA) are located nonrandomly with high abundance in the replication origin, D-loop, and stem-loop sequences [12]. While mitochondria are present in all eukaryotes, chloroplasts are exclusive for algae and plants. Therefore, it is thought that mitochondria were engulfed before the origin of chloroplasts by a common ancestor. This is also supported by the fact that mtDNA is usually shorter than cpDNA.

Chloroplasts are semiautonomous organelles; their origin dates back to over 1,000 million years ago, when an ancient cyanobacterium was engulfed by a eukaryotic cell (primary endosymbiotic event), which subsequently gave rise to glaucophytes, red algae, green algae, and plants [13] and probably most recently to the filose amoeba* Paulinella chromatophore* [14]. Subsequent secondary, serial secondary, and tertiary endosymbiotic processes have been important during the evolution of modern photosynthetic eukaryotes [15]. In land plants, cpDNA sequences are highly conserved and the genome can be divided into four different regions: large single copy (LSC) and small single copy (SSC), which are separated from each other by two large internal repeat regions. A cpDNA genome typically contains between 120 and 130 genes, mainly involved in photosynthesis, transcription, and translation. The cpDNA genome is usually between 107 kbp (*Cathaya argyrophylla*, Pinaceae family) and 218 kbp (*Pelargonium*, Geraniaceae family) in size. However, smaller cpDNA genomes are also common; for example, Apicomplexa genomes are around 30 kbp. On the other hand, the chromophore genome of* Paulinella chromatophora* is larger than some complete bacterial genomes, with 1* *022 kbp due to the unique recent engulfing event. Chloroplast genome size is independent of nuclear genome size [16].

The evolution of cpDNA genes is slower than that of nuclear genes [17], but faster than that of mtDNA genes [18]. Many of today's algae and almost all land plants carry two identical copies of a large coding sequence called the large IR A and IR B regions (varying from 20 kbp to 36 kbp), whose sequences may differ remarkably in individual species. In algae, the whole large IR has been lost multiple times during evolution [19]. In the parasitic and often nonphotosynthetic plants of Orobanchaceae family, the independent losses of one large IR region occur;* Conopholis americana* has the smallest cpDNA genome of land plants (45 kb) [20]. Loss of the large IR was also observed in some legumes (Papilionoideae subfamily, for example) [21]. Recently, it was found that, compared to single copy genes, synonymous substitution rates are on average 3.7-fold slower in chloroplast large IR genes (mainly coding for ribosomal, transfer RNAs, and ribosomal proteins). This may be due to the duplicative nature of the large IR, which reduces the substitution rate within these regions [22]. cpDNA rearrangements are more frequent when a large IR region is lost, suggesting that large IR regions are important for maintaining the conserved arrangement of cpDNA sequences [23]. In our analyses, we focused only on “short” IRs (6-60 bp) which are able to form cruciform structures. We analyzed these S-IRs in all sequenced cpDNA genomes to determine frequencies, localization, and similarities/differences.

## 2. Materials and Methods

### 2.1. Analyses of cpDNA Sequences

The set of 2,566 complete plastid cpDNA sequences were downloaded from the genome database of the National Center for Biotechnology Information (NCBI). We used the computational core of our DNA analyser software [24] modified to read NCBI identifiers of sequences from a text file and to download them. The parameters of analyses were size of S-IR 6 to 60 bp and spacer size 0 to 10 bp, and maximally one mismatch was allowed. Analysis produced a separate list of S-IRs found in each of the 2,566 cpDNA sequences and an overall report for each selected group. Overall results for each species group contained a list of species with the size of its cpDNA sequence and the number of S-IRs found in that sequence. Our software also counted S-IRs grouped by their individual size (6–60 bp individually and the sum of S-IRs longer than 8 bp, 10 bp, and 12 bp).

### 2.2. Analysis of S-IRs around Annotated NCBI Features

We downloaded the genome feature tables from the NCBI database along with the cpDNA sequences. We performed analysis of S-IR occurrence inside and around (before and after) recorded features. Features were grouped by their name stated in the feature table file. From this analysis we obtained a file with feature names and numbers of S-IRs found inside and around features for each group of species analyzed. Search for S-IRs took place in predefined feature neighborhoods (we used ±100 bp; this figure is important for calculation of S-IR frequency in feature neighborhood) and inside feature boundaries. We calculated the amount of all S-IRs and those longer than 8, 10, and 12 bp in regions before, inside, and after features. The categorization of an S-IR according to its overlap with a feature or feature neighborhood is demonstrated by the example shown in Supplementary Figure S1. Further processing was performed in Microsoft Excel.

### 2.3. Analyses of Similarities

Similarity among S-IRs was performed for those with abundant presence in the cpDNA genomes by Levenshtein algorithm, which counts distance between two strings according to the number of deletions, insertions, or substitutions required to transform source string into target string [25].

### 2.4. Phylogenetic Tree Construction

Exact taxid IDs of all analyzed groups (obtained from Taxonomy Browser via NCBI Taxonomy Database [26]) were downloaded to phyloT: a tree generator (http://phylot.biobyte.de) and a phylogenetic tree was constructed using function “Visualize in iTOL” in Interactive Tree of Life environment [27]. The resulting tree is shown in Supplementary Figure 2.

### 2.5. Statistical Analysis

Cluster dendrogram of S-IR frequency data (Supplementary Table S1) was made in R v. 3. 4. 3 (R Core Team, 2016) using the hclust function with the cluster method “ward.D2”. The resulting cluster dendrogram is shown in Supplementary Figure S2. Principal component analysis (PCA) interactive plots were made in R with ggplot2 [28] and plotly [29]. The R code is available in Supplementary Code S1. Frequency of S-IRs (categorized by length) in individual species groups was used as input data, so one PCA plot was constructed for each species group to display intragroup variability.

## 3. Results and Discussion

### 3.1. General Statistics for cpDNA

**Figure 1:**
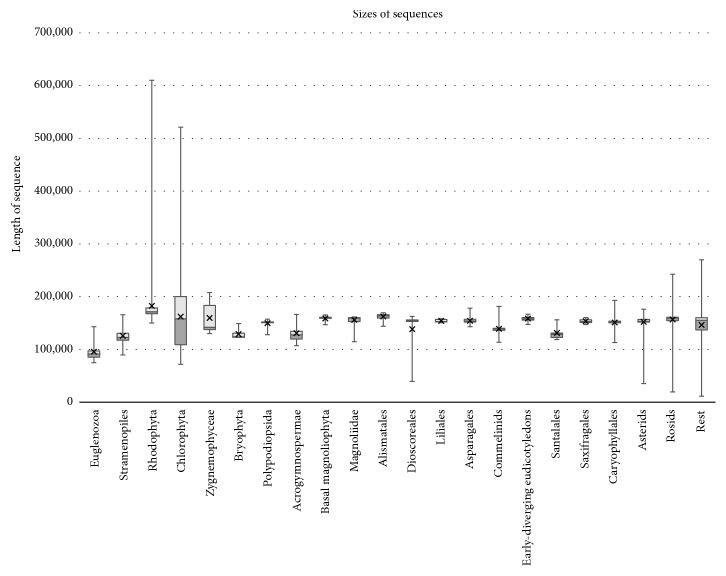
Variability of length of cpDNAs. Box plots show sequence length interquartile ranges for different species groups. The whiskers represent the minimum and maximum values.

cpDNAs are stored in the genome database in the three taxonomy groups (Protists, Plants, and others) and four subgroups (Apicomplexans, Green Algae, Plants, and others). However, the vast majority of sequences belong to the Plants subgroup (2,278), compared to Apicomplexans (36) and Green Algae (107). Due to discrepancies in the number of sequenced cpDNA genomes in diverse groups (for example, the phylogenetically important group Euglenozoa has only 9 sequenced cpDNAs whereas Rosids and Asterids in the Pentapalae group [30] each have more than 400), we divided downloaded sequences into 21 phylogenetically related groups with a minimum of eight members in each group (see Supplementary Figure 2). This division allows us to observe detailed trends in S-IR frequency evolution across the Plant kingdom (from evolutionary oldest Bryophyta to Polypodiopsida, Acrogymnospermae, Basal Magnoliophyta, Magnoliidae, Alismatales, Dioscoreales, Liliales, Asparagales, Commelinids, Early-Diverging Eudicotyledons, Santalales, Saxifragales, Caryophyllales, to Asterids and Rosids). In total, we have analyzed 2,565 plastid genomes and, in addition, the chromatophore genome of amoeba* Paulinella chromatophora*. The length of cpDNA sequences (Table 1) varies from 11,348 bp (*Pilostyles aethiopica, *endoparasitic land plant which preserved only 17 chloroplast genes of the usual number of 116 cpDNA genes [16]) to 610,063 bp in* Bulboplastis apyrenoidosa* algae from the Rhodophyta division. Recently, a plastid DNA was described for red algae* Corynoplastis japonica* as the largest and most intron-rich plastid genome (1.13 Mbp) [31]. However, the majority of cpDNA genomes varied in the close interval between 120 and 150 kbp. The cpDNA genome of* Paulinella chromatophore*, which has a plastid genome from a different engulfment event estimated only 60 million years ago, is 1,021,616 bp [14], longer than the whole genomes of individual prokaryotic organisms. Aside from this unique organism, the longest cpDNAs are typical for Rhodophyta, Chlorophyta, and Zygnemophyceae, and the shortest for unicellular organisms in the phylum Euglenozoa (Figure 1). Length variability is generally correlated with evolutionary age. The largest variability is observed in the group Chlorophyta and Rhodophyta while the sizes of cpDNA in group Liliales and other land plants are relatively constant. The variability of length in higher plants is limited compared to lower plants (bryophyta, algae, etc.) (Figure 1). Contrary to the diverse sizes of cpDNA in ancient phylogenetic groups, the size of the cpDNA in phylogenetically newer groups is more limited with only a few exceptions. For example, 75% of all 522 sequenced cpDNA genomes in clade Rosids are in the size interval of 159,441 to 160,886 bp, differing by less than 1%.

### 3.2. Analyses of Short Inverted Repeats

**Figure 2:**
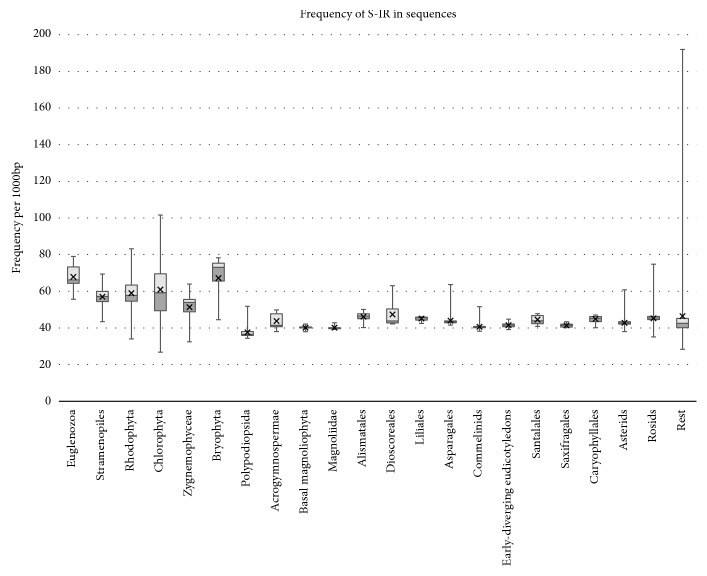
Frequency of S-IRs in mtDNAs for subgroups and numbers of mtDNAs. The box plot shows the interquartile ranges of S-IR frequencies per 1000 bp in different species groups. Whiskers represent the minimum and maximum values.

The total number of nucleotides in the 2,566 plastid genomes analyzed is 384,975,139 bp and we found 17,326,953 S-IRs. The average frequency is 47 (17-81) S-IR/kbp for green algae and 34 (29-59) S-IR/kbp for land plants. The differences between organisms are significant; 50% of cpDNAs have a frequency of 40 to 45 S-IR/kbp, but S-IR frequencies range from 26 S-IR/kbp in unicellular green algae of the order Mamiellales* Ostreococcus tauri*, while other green algae, specifically of the order Volvocaceae* Pleodorina starrii* have a frequency of 191.98 S-IR/kbp.* Ostreococcus tauri* is a member of global oceanic picoplankton and is the smallest described free-living eukaryote with very a compact genome.* Pleodorina starrii *is another alga which is composed of 32 or 64 biflagellate cells. Values of S-IR frequencies for all groups are shown in Figure 2. The highest S-IR frequencies are in the groups Euglenozoa (67.87 S-IR/kbp) and Bryophyta (67.24 S-IR/kbp) followed by Chlorophyta (60.95 S-IR/kbp) and Rhodophyta (59 S-IR/kbp) and the lowest S-IR frequencies are in the groups Polypodiopsida (37.65 S-IR/kbp) followed by Basal Magnoliophyta (40.11 S-IR/kbp), Magnoliidae (40.14 S-IR/kbp), and Commelinids (40.63 S-IR/kbp). Statistics and evaluation for all groups are provided in Supplementary Table S2.

Comparing S-IRs in individual organisms and subgroups shows a general decrease in frequency with increasing S-IR length, except for S-IRs 15, 22, 24, or 27 bp long, which are present more often than expected by approximation from neighboring values (Table 1). Similar selective abundance according to length has also been observed for mtDNAs, but only for S-IR lengths 24 and 30 bp. Both 24 and 30 bp long S-IRs are also more frequent than expected in cpDNA sequences, but there is very strong relative abundance also for 15, 22, and 27 bp S-IRs. We investigated if the S-IRs abundantly present in cpDNA genomes are similar using analyses of similarity by Levenshtein distance (Supplementary Data 1). We used two different conditions for the comparison, identity and Levenshtein distance 2 or less. These results show that especially longer S-IR sequences are shared only for a few phylogenetic groups; on the other hand shorter S-IR sequences are often shared across several phylogenetic groups (Supplementary Data 1). Our results show that 27 bp S-IRs are present most often as the sequences AAATTCTTTTATTTTAGATAGAAGAAA and ACATTCTTTTATTTTAGATAGAAGAAA (both 320 times); the next most abundant sequence was present in all genomes only 34 times. Both sequences are identical except for one nucleotide; therefore for Levenshtein distance 2 and less we found AAATTCTTTTATTTTAGATAGAAGAAA sequence 655 times, followed by TATAAGTGAACTAGATAAAACGGAATC sequence 49 times. For 22 bp long S-IRs, the sequence AGAGCTCGGATCGAATCGGTAT is present 414 times; the next most abundant sequence was present only 89 times in all genomes. For Levenshtein distance 2 and less we found AGAGCTCGGATCGAATCGGTAT sequence in 590 cases, followed by TAATTGAAGTAAGAAGTCTCCC sequence 240 times. For 15 bp long S-IRs, the most common sequences were ATAAAAGAAAGAAGA and AAAAAAGAAAGAAGA (presented 1,346 and 1,341 times, respectively), and the next most abundant sequence was present only 370 times in all genomes. Both most abundant sequences are identical except for one nucleotide; therefore for Levenshtein distance 2 and less we found ATAAAAGAAAGAAGA 2,702 times, followed by AAAAAAAAAGAAAGA 1,272 times.

The detailed results of S-IR frequencies for all groups are summarized in Table 2. The most common longest S-IRs varied from 17 bp (in Commelinids) to 32 bp (in Bryophyta). The most common longest S-IR in vascular plants (Tracheophyta) (24 bp) was found in Asparagales. We also found 1,105 S-IRs longer than 30 bp in cpDNA genomes (range 31-100 bp), but they constituted less than 0.01% of the total number of 17,326,953 S-IRs identified.

### 3.3. Comparison of S-IR Frequencies according to Sequence Annotations

**Figure 3:**
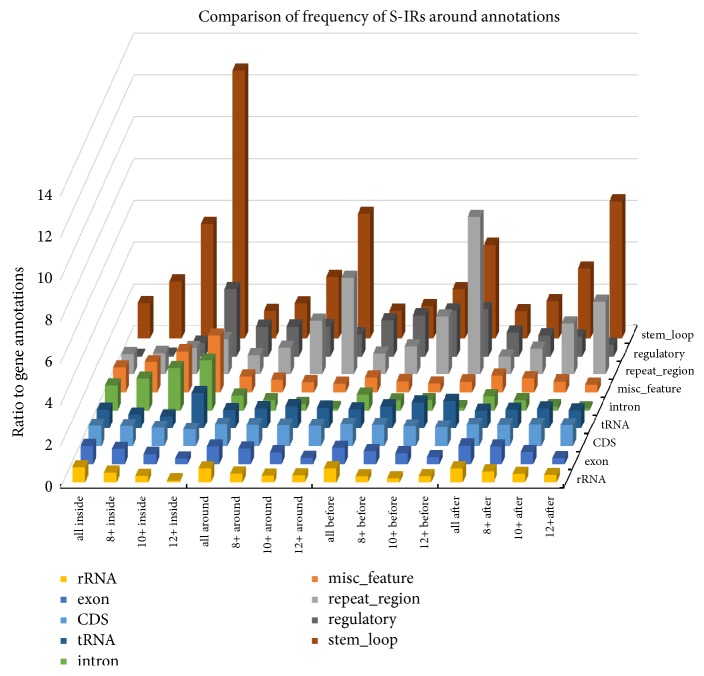
Differences in S-IR frequency by DNA locus. The chart shows S-IR frequencies per 1000 bp between “gene” annotation and other annotated locations from the NCBI database. We analyzed frequencies of all S-IRs (all) and of S-IRs with lengths 8 bp and longer (8+), 10 bp and longer (10+), and 12 bp and longer (12+) within annotated locations (inside) and before (100 bp) and after (100 bp) annotated locations.

The NCBI genome database contains annotations for cpDNA sequences. The best described are gene (343,857), CDS (226,783), tRNA (91,586), exon (36,345), rRNA (18,719), and intron (11,028). Numbers of annotations at the time of analysis are given in Supplementary Table S3. To compare S-IR frequencies at different locations we used the most commonly described location “gene” as a standard for comparison with other locations. There are significant differences in S-IR frequency in diverse segments of cpDNAs. The largest relative increase of S-IR frequency is for stem-loop sequences (12.8-times higher) followed by regulatory (3.2x), intron (2.4x), and misc sequences (2.7x) (Figure 3). The presence of the “large internal repeat region” is typical for higher plants. The difference between S-IR frequency in the large internal repeat regions and gene regions is only 1.7-fold, whereas S-IRs are 4.6-fold higher in the sequences surrounding these features and the frequency of 12 bp S-IRs and longer is 7.6 times higher before the repeat region compared to gene regions. Another interesting finding is the presence of S-IRs in introns; while S-IRs are abundant in introns, S-IR frequencies are significantly reduced in exon sequences that neighbor introns (both 100 bp before and after introns). We observed significantly reduced S-IR frequencies in and around rRNAs compared to gene locations, especially for 12 bp and longer S-IRs. The opposite effect is seen for tRNA sequences. Raw data are in Supplementary Tables S4 and S5.

Based on the data from S-IR analyses we produced a cluster dendrogram of individual groups (Supplementary Figure 2). The real phylogenetic tree is fairly consistent with this cluster dendrogram. Very interesting is the proximity of Euglenozoa and Bryophyta clades. However, this may be due the fact that both groups contain only a small number of accessible sequenced genomes (only 9 and 8 cpDNA genomes are available for Euglenozoa and Bryophyta, respectively).

Based on PCA analysis (Supplementary Data 2.html) of S-IR frequencies data (Supplementary Table S2), some interesting facts emerged. The most interesting findings in individual groups from our point of view are shortly commented with appropriate references in the following bullet paragraphs; for details please check interactive graphs (Supplementary Data 2.html):

- In Rosids, the cpDNA of the holoparasitic plant* Cytinus hypocistis *was sequenced recently [20] and PCA analysis of S-IR frequency revealed a very unusual profile in comparison with the rest of the group (Supplementary Data 2.html).* Cytinus hypocistis* has an extremely small cpDNA genome (19.4 kb) with only 23 genes and no large IR-A or IR-B regions [20]. On the other hand,* Cytinus hypocistis *has the highest S-IR/kbp cpDNA value from all 522 analyzed Rosids (78.81 S-IR/kbp).
- In Asterids, the most divergent S-IR frequencies in cpDNA include two species of the family Orobanchaceae (*Phelipanche ramosa* and* Phelipanche purpurea*), which are nonphotosynthetic flowering plants. Their S-IR frequencies are unusually high in comparison to the rest of the Asterids.
- In Caryophyllales, the flytrap* Dionaea muscipula *has an unusually low S-IR frequency in cpDNA.
- In Early-Diverging Eudicotyledons,* Gymnospermium microrrhynchum *has the highest S-IR frequency, especially for S-IRs longer than 12 bp.
- In Commelinids,* Carex neurocarpa* has the highest S-IR frequency in cpDNA. In Asparagales,* Cypripedium formosanum* (endemic orchid of Taiwan) has a very high S-IR frequency in cpDNA.
- In Liliales, all three PCA clusters are very well distinguished from each other. In Dioscoreales,* Burmannia oblonga* has the highest S-IR frequency in cpDNA.
- In Alismatales, the aquatic plant* Najas flexilis* has a low S-IR frequency in cpDNA in comparison to the rest of the group. The plastid genome of* Najas flexilis* presents some anomalous modifications (reduced size of a small single copy region, eleven* ndh* gene losses) [21].
- In Magnoliidae,* Cassytha filiformis* and two species of pepper vine* (Piper kadsura* and* Piper cenocladum*) have the most divergent pattern of S-IR in cpDNA (higher frequencies of S-IRs) compared with the rest of group.
- In Basal Magnoliophyta,* Trithuria inconspicua* (endemic aquatic herb of New Zealand) has the lowest S-IR frequency in cpDNA in comparison with the rest of the group (especially for S-IRs longer than 10 bp).
- In acrogymnospermae, all three PCA clusters are very well distinguished from each other (Supplementary Data 2). In Polypodiopsida, two species of class Equisetopsida (*Equisetum hyemale* and* Equisetum arvense*) have a very high frequency of S-IRs compared with other species in this group.
- In Bryophyta, two species (*Sphagnum palustre* and* Takakia lepidozioides*) have very low S-IR frequencies in cpDNA in comparison with the rest of the group.
- In Zygnemophyceae,* Spirogyra maxima *has the highest frequency of S-IR in cpDNA (for S-IRs longer than 12 bp there is more than 10-fold enrichment in comparison with the other species in the group).
- In Chlorophyta, four species of family Ulvaceae (*Ulva fasciata*,* Ulva linza*,* Ulva prolifera,* and* Ulva flexuosa*) have very high frequencies of S-IRs in comparison with the rest of the group.
- In Rhodophyta, the strangest pattern of S-IR frequency in cpDNA was found in* Cyanidioschyzon merolae*, a small unicellular haploid red alga adapted to hot spring environment with high sulfur acidic content.* Cyanidioschyzon merolae* has only one chloroplast and mitochondria. After detailed analysis we have found that cpDNA of* Cyanidioschyzon merolae* has an extremely low content of S-IRs in comparison to the rest of the Rhodophyta group.
- In Stramenopiles,* Aureococcus anophagefferens* (unicellular alga with only one chloroplast and mitochondrion) and* Aureoumbra lagunensis* have the lowest S-IR frequency in cpDNA in comparison with the rest of the group. In* Aureococcus anophagefferens* and* Aureoumbra lagunensis* the large IR regions were lost [22].
- In Euglenozoa,* Monomorphina aenigmatica* has the highest S-IR frequency and the plastid genome of this species is AT rich (70.6%) and contains 53 intron insertion sites, of which 41 were found to be shared with other euglenids [23].

DNA cruciforms are formed by S-IRs and with their important roles in replication, transcription and DNA stability it is not surprising that S-IRs are also present in cpDNA genomes. Analyses of mtDNA genomes revealed that S-IR sequences are abundant and nonrandomly distributed in the mitochondrial genomes of all living organisms, with particular abundance in regulatory regions such as replication origin and D-loop region [12]. Here, we analyzed all available cpDNA genomes for the presence of S-IRs capable of forming cruciform structures. Our results show that the typical maximal S-IR length in cpDNA varied from 17 bp (Commelinids) to 32 bp (Bryophyta). Interestingly, substantial numbers of longer S-IRs are detected in some cpDNAs. While the mean frequency of S-IR was ~45 IRs/kbp, the frequency was remarkably higher in some plastid genomes. In 13 plastid genomes, the frequency was higher than 100 S-IR/kbp, most of these genomes were from Apicomplexa, but also red algae* Choreocolax polysiphoniae*, which has reduced gene regions [32]. These organisms typically contain an AT-rich relict plastid which functions in fatty acid biosynthesis, heme biosynthesis, iron-sulfur cluster synthesis, and isoprenoid biosynthesis, but they are deficient in photosynthetic abilities [33]. Long IRs (about 20-36 kbp long) in cpDNA can help stabilize the rest of the chloroplast genome and prevent gene loss-and-gain rearrangements [34]. Interestingly, these longer IRs are surrounded by short S-IRs. While the difference between S-IR frequency in the repeat regions and gene regions is relatively small, the frequency of 12 bp and longer S-IRs in the surrounding sequences is 7.6 times higher before the repeat region. It is likely that S-IRs in cpDNA also form cruciform structures in vivo and these are targets for binding of specific proteins (our hypothesis is based on cruciform functions and preferential protein binding to cruciforms in human and other model organisms). Some information is available to support this concept in plants; ribosomal protein S16 (RPS16) binds to cruciform DNA and is targeted to the chloroplast, indicating the possibility of RPS16-cruciform cpDNA interactions and regulation [35]. Moreover, S-IRs were found to be highly enriched in stem-loop sequences, which have an important regulatory function in cpDNAs.

## 4. Conclusions

Chloroplasts, as basic organelles for algae and plants, are fundamental for life due to their oxygen management. In this paper, we analyzed all 2,566 sequenced cpDNA by “palindrome analyser”. We described the basic parameters of cpDNA including the frequency and localization of S-IRs able to form cruciform structures. Interestingly, the frequency of S-IR does not decrease for S-IRs 15, 22, 24, or 27 bp long. These results point to the importance of specific S-IRs in cpDNA genomes. Moreover, comparison by Levenshtein distance of S-IR similarities showed that a limited number of sequences are shared in the majority of cpDNA S-IRs. S-IRs are not located randomly, but are length-dependently enriched in specific locations, including the repeat region, stem, introns, and tRNA regions of cpDNA genomes. The highest enrichment was found for 12 bp and longer S-IRs in the stem-loop region, followed by 12 bp and longer S-IRs located before the repeat region. These data showing nonrandom and conserved arrangements of S-IRs in chloroplast genomes indicate the potential importance of S-IRs in basic biological processes within chloroplasts.

## Figures and Tables

**Table 1:** Numbers and frequencies of S-IRs according to size.

IR size	Amount in dataset	IR frequency per 1000bp	IR size	Amount in dataset	IR frequency per 1000bp	IR size	Amount in dataset	IR frequency per 1000bp
6	10,351,040	26.899	15	13,370	0.035	24	1,619	0.004

7	4,157,127	10.803	16	6,641	0.017	25	1,005	0.003

8	1,656,101	4.304	17	5,505	0.014	26	760	0.002

9	637,184	1.656	18	3,595	0.009	27	1,231	0.003

10	264,249	0.687	19	2,783	0.007	28	450	0.001

11	113,649	0.295	20	2,676	0.007	29	350	0.001

12	50,229	0.131	21	2,108	0.005	30	302	0.001

13	27,833	0.072	22	3,090	0.008	>30	1,105	0.004

14	13,935	0.036	23	1,577	0.004			

**Table 2:** cpDNA sizes and S-IR frequencies and lengths.

Group name	Number of seq.	Median size [bp]	Shortest sequence	Longest sequence	IR/kbp Mean range	Longest S-IR for 50% of seq. [bp]
**Euglenozoa**	9	91,616	Monomorphina aenigmatica	Euglena gracilis	68	18
			(74,746 bp)	(143,171 bp)	56 – 79	
**Stramenopiles**	37	122,660	Aureococcus anophagefferens	Cylindrotheca closterium	57	25
			(89,599 bp)	(165,809 bp)	43 – 69	
**Rhodophyta**	60	171,284	Cyanidioschyzon merolae	Bulboplastis apyrenoidosa	59	19
			(149,987 bp)	(610,063 bp)	34 -83	
**Chlorophyta**	90	157,916	Ostreococcus tauri	Floydiella terrestris	61	27
			(71,666 bp)	(521,168 bp)	27 – 102	
**Zygnemophyceae**	11	142,017	Spirogyra maxima	Cosmarium botrytis	51	24
			(129,954 bp)	(207,850 bp)	32 – 64	
**Bryophyta**	8	123,868	Syntrichia ruralis	Takakia lepidozioides	67	32
			(122,630 bp)	(149,016 bp)	44 – 78	
**Polypodiopsida**	49	151,126	Diplazium unilobum	Lygodium japonicum	38	18
			(127,840 bp)	(157,260 bp)	34 – 52	
**Acrogymnospermae**	85	127,659	Cathaya argyrophylla	Macrozamia mountperriensis	44	23
			(107,122 bp)	(166,341 bp)	38 – 50	
**Basal magnoliophyta**	13	159,881	Schisandra chinensis	Trithuria inconspicua	40	18
			(146,859 bp)	(165,389 bp)	38 – 42	
**Magnoliidae**	41	159,443	Cassytha filiformis	Piper kadsura	40	18
			(114,622 bp)	(161,486 bp)	39 – 43	
**Alismatales**	14	163,856	Zostera marina	Wolffiella ryophyte	46	22
			(143,877 bp)	(169,337 bp)	40 – 50	
**Dioscoreales**	10	154,205	Burmannia oblonga	Tacca leontopetaloides	47	22
			(39,386 bp)	(162,477 bp)	42 – 63	
**Liliales**	41	152,677	Amana wanzhensis	Heloniopsis tubiflora	45	18
			(150,576 bp)	(158,229 bp)	42 – 46	
**Asparagales**	125	153,953	Oberonia japonica	Cypripedium formosanum	44	24
			(142,996 bp)	(178,131 bp)	42 – 64	
**Commelinids**	290	139,171	Aegilops cylindrica	Carex neurocarpa	41	17
			(113,490 bp)	(181,397 bp)	38 – 52	
**Early-diverging eudicotyledons**	49	157,817	Kingdonia uniflora	Berberis koreana	42	19
			(147,378 bp)	(166,758 bp)	39 – 45	
**Santalales**	9	128,744	Schoepfia jasminodora	Erythropalum scandens	45	18
			(118,743 bp)	(156,154 bp)	41 – 48	
**Saxifragales**	10	152,692	Phedimus takesimensis	Liquidambar formosana	41	20
			(147,048 bp)	(160,410 bp)	40 – 43	
**Caryophyllales**	32	151,686	Carnegiea gigantea	Drosera rotundifolia	45	19
			(113,064 bp)	(192,912 bp)	40 – 47	
**Asterids**	398	153,377	Monotropa hypopitys	Adenophora divaricata	43	19
			(35,336 bp)	(176,331 bp)	38 – 61	
**Rosids**	522	159,441	Cytinus hypocistis	Pelargonium transvaalense	45	20
			(19,400 bp)	(242,575 bp)	35 – 75	
**Rest**	662	155,196	Pilostyles aethiopica	Pleodorina starrii	46	20
			(11,348)	(269,857)	28 – 192	

## Data Availability

All data are freely accessible in the paper and in supporting materials.

## Supplementary Materials

Supplementary Material Supplementary Figure S1: neighborhood of an annotated feature. Example of possible S-IR occurrence around features and its classification: (a) an S-IR overlapping only partially with a feature is considered to be in near neighborhood; (b) an S-IR overlapping fully with a feature is therefore considered to be inside; (c) an S-IR is not considered to be in near neighborhood because it is not fully overlapping with either a feature or its neighborhood. Supplementary Figure 2: phylogenetic tree of all inspected organisms with chloroplast genome made using iTOL. Subgroups are highlighted by different colors. From left counterclockwise: Rosids (red, 522 species); Asterids (blue, 398 species); Caryophyllales (dark green, 32 species); Saxifragales (yellow, 10 species); Santalales (purple, 9 species); Early-Diverging Eudicotyledons (green, 49 species); Commelinids (red, 290 species); Asparagales (blue, 125 species); Liliales (yellow, 41 species); Dioscoreales (purple, 10 species); Alismatales (dark green, 14 species); Magnoliidae (orange, 41 species); Basal Magnoliophyta (green, 13 species); Acrogymnospermae (red, 85 species); Polypodiopsida (green, 49); Bryophyta (orange, 8 species); Zygnemophyceae (red, 11 species); Chlorophyta (purple, 90 species); Rhodophyta (green, 60 species); Stramenopiles (orange, 37 species); Euglenozoa (blue, 9 species). Supplementary Code S1: method for construction of interactive PCA plots from S-IR data by R (version 3.4.0). Referred Excel input for this analysis was values from even S-IR length columns of Supplementary Table S1. Supplementary Table S1: incidence of S-IRs. This table represents ratio of presence of S-IRs by their length. Values were calculated by the following formula: number of sequences containing at least one S-IR of given length in a subgroup/total number of sequences in a subgroup. For example, in Alismatales subgroup, there is a total of 14 S-IR sequences, 9 of those sequences have S-IRs of length 24, and thus 9 / 14 = 0.64. Supplementary Table S2: statistical evaluation of results. This table contains statistical data about groups of sequences. Row denoted usual longest S-IR contains length of S-IR that is present in roughly half of the sequences of that group; see Table S3. Supplementary Table S3: feature amounts and length. This table shows amounts of annotated features in all downloaded sequences and their length. Supplementary Data 1: analysis of S-IR similarity for lengths 15, 22, 24, and 27. The first four sheets group identical S-IRs from all cpDNA sequences. The last four sheets group S-IRs that are similar, based on Levenshtein distance of 2 or less. The upper part of each sheet contains the 20 most abundant S-IRs identified by rank. Lower parts of the sheets contain incidence for given S-IRs by rank in different groups of species. Supplementary Data 2.html: PCA plots: interactive PCA plots intuitively represent differences in cpDNA S-IR frequencies of all main groups and intradifferences of cpDNA S-IR frequencies between organisms of each subgroup. Organisms with the most distinct patterns of S-IR frequencies in their cpDNA are always more distant from the middle of the plot.
