# Supplementary material for: Complex Analyses of Short Inverted Repeats in All Sequenced Chloroplast DNAs
Source: Biomed Res Int. 2018 Jul 24;2018:1097018. doi: 10.1155/2018/1097018 (PMC6081594; doi:10.1155/2018/1097018)
Supplement: Supplementary Material — Supplementary Figure S1: neighborhood of an annotated feature. Example of possible S-IR occurrence around features and its classification: (a) an S-IR overlapping only partially with a feature is considered to be in near neighborhood; (b) an S-IR overlapping fully with a feature is therefore considered to be inside; (c) an S-IR is not considered to be in near neighborhood because it is not fully overlapping with either a feature or its neighborhood. Supplementary Figure 2: phylogenetic tree of all inspected organisms with chloroplast genome made using iTOL. Subgroups are highlighted by different colors. From left counterclockwise: Rosids (red, 522 species); Asterids (blue, 398 species); Caryophyllales (dark green, 32 species); Saxifragales (yellow, 10 species); Santalales (purple, 9 species); Early-Diverging Eudicotyledons (green, 49 species); Commelinids (red, 290 species); Asparagales (blue, 125 species); Liliales (yellow, 41 species); Dioscoreales (purple, 10 species); Alismatales (dark green, 14 species); Magnoliidae (orange, 41 species); Basal Magnoliophyta (green, 13 species); Acrogymnospermae (red, 85 species); Polypodiopsida (green, 49); Bryophyta (orange, 8 species); Zygnemophyceae (red, 11 species); Chlorophyta (purple, 90 species); Rhodophyta (green, 60 species); Stramenopiles (orange, 37 species); Euglenozoa (blue, 9 species). Supplementary Code S1: method for construction of interactive PCA plots from S-IR data by R (version 3.4.0). Referred Excel input for this analysis was values from even S-IR length columns of Supplementary Table S1. Supplementary Table S1: incidence of S-IRs. This table represents ratio of presence of S-IRs by their length. Values were calculated by the following formula: number of sequences containing at least one S-IR of given length in a subgroup/total number of sequences in a subgroup. For example, in Alismatales subgroup, there is a total of 14 S-IR sequences, 9 of those sequences have S-IRs of length 24, and thus 9 / 14 = 0.6 [file 1097018.f1.zip › Supplementary data 2.html]

Supplementary plots


# Supplementary plots

PCA interactive plot of IRs incidence pattern for all subgroups of analyzed organisms.

By default, data was always assorted into three colored clusters for better orientation and pointing up differences.

Interactive PCA plots intuitively represent differences in cDNA IR frequencies of all main groups and intra-differences
of cDNA IR frequencies between organisms of each subgroup. Organisms which the most unique patterns of IR frequencies
in their cDNA are always more distant from the middle of the plot.

- P1 - All species
- P2 - Euglenozoa
- P3 - Stramenopiles
- P4 - Rhodophyta
- P5 - Chlorophyta
- P6 - Zygnemophyceae
- P7 - Bryophyta
- P8 - Polypodiopsida
- P9 - Acrogymnospermae
- P10 - Basal magnoliophyta
- P11 - Magnoliidae
- P12 - Alismatales
- P13 - Dioscoreales
- P14 - Liliales
- P15 - Asparagales
- P16 - Commelidins
- P17 - Early-diverging eudicotyledons
- P18 - Santalales
- P19 - Saxifragales
- P20 - Caryophyllales
- P21 - Asterids
- P22 - Rosids
