# Supplementary material for: Complex Analyses of Short Inverted Repeats in All Sequenced Chloroplast DNAs
Source: Biomed Res Int. 2018 Jul 24;2018:1097018. doi: 10.1155/2018/1097018 (PMC6081594; doi:10.1155/2018/1097018)
Supplement: Supplementary Material — Supplementary Figure S1: neighborhood of an annotated feature. Example of possible S-IR occurrence around features and its classification: (a) an S-IR overlapping only partially with a feature is considered to be in near neighborhood; (b) an S-IR overlapping fully with a feature is therefore considered to be inside; (c) an S-IR is not considered to be in near neighborhood because it is not fully overlapping with either a feature or its neighborhood. Supplementary Figure 2: phylogenetic tree of all inspected organisms with chloroplast genome made using iTOL. Subgroups are highlighted by different colors. From left counterclockwise: Rosids (red, 522 species); Asterids (blue, 398 species); Caryophyllales (dark green, 32 species); Saxifragales (yellow, 10 species); Santalales (purple, 9 species); Early-Diverging Eudicotyledons (green, 49 species); Commelinids (red, 290 species); Asparagales (blue, 125 species); Liliales (yellow, 41 species); Dioscoreales (purple, 10 species); Alismatales (dark green, 14 species); Magnoliidae (orange, 41 species); Basal Magnoliophyta (green, 13 species); Acrogymnospermae (red, 85 species); Polypodiopsida (green, 49); Bryophyta (orange, 8 species); Zygnemophyceae (red, 11 species); Chlorophyta (purple, 90 species); Rhodophyta (green, 60 species); Stramenopiles (orange, 37 species); Euglenozoa (blue, 9 species). Supplementary Code S1: method for construction of interactive PCA plots from S-IR data by R (version 3.4.0). Referred Excel input for this analysis was values from even S-IR length columns of Supplementary Table S1. Supplementary Table S1: incidence of S-IRs. This table represents ratio of presence of S-IRs by their length. Values were calculated by the following formula: number of sequences containing at least one S-IR of given length in a subgroup/total number of sequences in a subgroup. For example, in Alismatales subgroup, there is a total of 14 S-IR sequences, 9 of those sequences have S-IRs of length 24, and thus 9 / 14 = 0.6 [file 1097018.f1.zip › Supplementary material.docx]

| **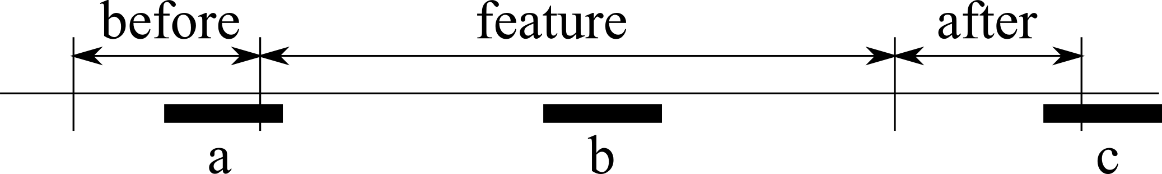** |
| --- |
| Supplementary Figure S1: Neighborhood of an annotated feature. Example of possible IR occurrence around features and its classification: a) An inverted repeat is considered to be in near neighbourhood because it overlaps only partially with a feature. b) An inverted repeat overlapping fully with a feature and therefore is considered to be inside. c) An inverted repeat is not considered to be in near neighbourhood because it is not fully overlapping neither with a feature or its neighbourhood. |
| 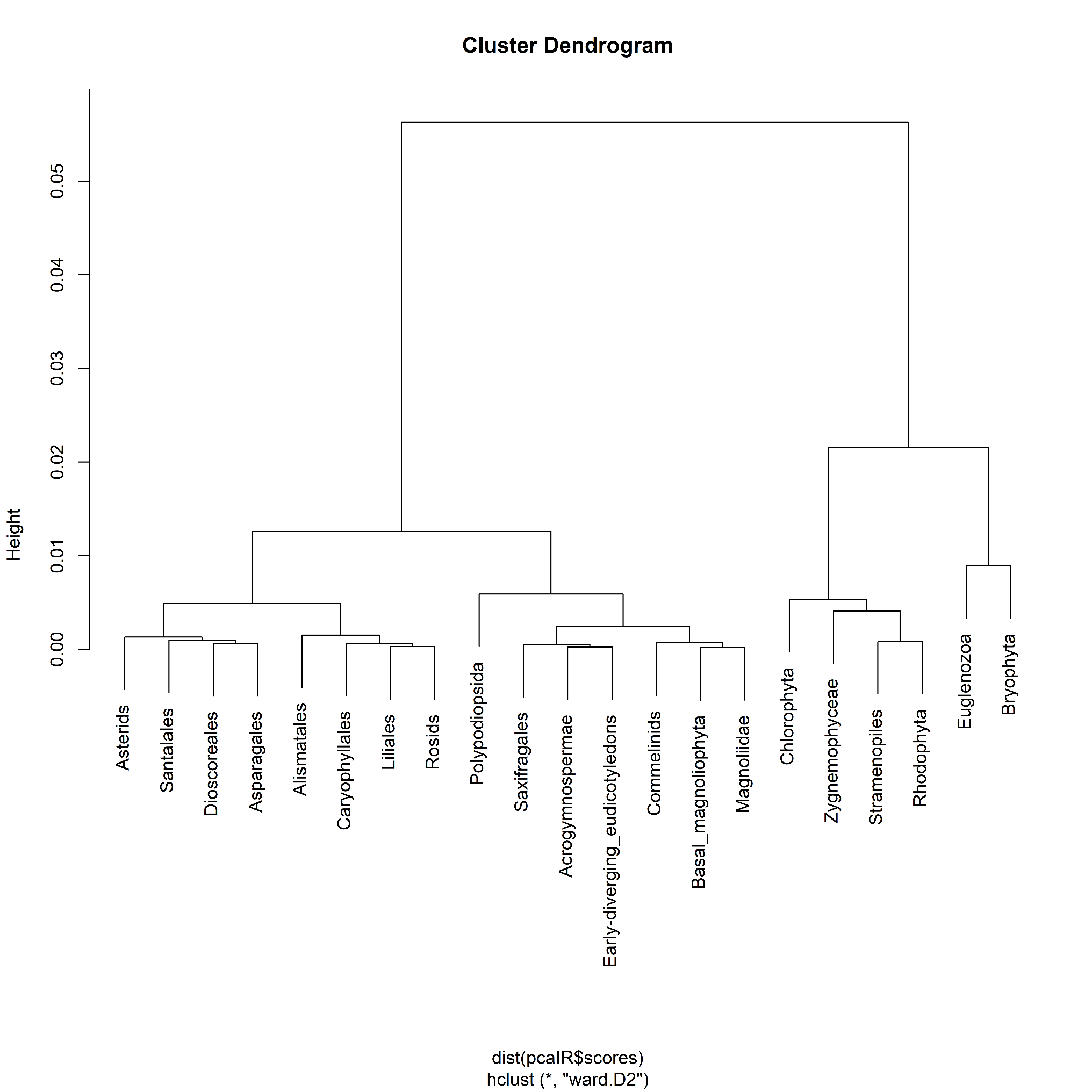 |
| Supplementary Figure S2: Cluster dendrogram of analysed groups of organisms based on inverted repeats incidence in their mitochondrial genomes. Dendrogram was constructed in R using 10 000 bootstrap replications with the cluster method “average”. Red au values indicate support of each branching (above 95 is considered to be very well supported). Height of the dendrogram (y-axis) shows relative distinctness of particular groups from the others. It is clearly visible, that the cluster dendrogram doesn´t copy real phylogenetic tree at all. On the other side, some phylogenetic close groups, for example amphibians, reptiles (altought polyphyletic) and birds are on the same branch as well as green algae and land plants. Based on this data (incidence of inverted repeats in mitochondrial genomes), most distinct group from the others are apicomplexans. |
| x<-read.table("clipboard",h=T,dec=",") #loading data from Excel by Ctrl+C, Ctrl+V  pcaIR<-princomp(x) #transforming data into principal comp.  IRHC<-hclust(dist(pcaIR$scores),method="ward.D2") #dendrogram  plot(IRHC) #plot dendrogram  IRclusters<-cutree(IRHC,k=3) # k for number of clusters  IRDf<-data.frame(pcaIR$scores,"cluster"=factor(IRclusters))  IRDf<-transform(IRDf,cluster_name=paste("Cluster",IRclusters))  library(ggplot2) #library must be previously installed  library(plotly) #library must be previously installed  p<-plot_ly(IRDf,x=IRDf$Comp.1, y=IRDf$Comp.2, text=rownames(IRDf), mode="markers", color=IRDf$cluster_name, marker=list(size=11))  p<-layout(p,title="PCA Clusters from Hierarchical Clustering of IR Data", xaxis=list(title="PC 1"), yaxis=list(title="PC 2"))  p #final plot constructaion |
| Supplementary Code S1: Method for construction of interactive PCA plots from IR data by program R (version 3.4.0). Referred Excel input for this analysis was essentially values from even IR length columns of Supplementary Table S1. |

|  | 6 | 7 | 8 | 9 | 10 | 11 | 12 | 13 | 14 | 15 | 16 | 17 | 18 | 19 | 20 | 21 | 22 | 23 | 24 | 25 | 26 | 27 | 28 | 29 | 30 |
| --- | --- | --- | --- | --- | --- | --- | --- | --- | --- | --- | --- | --- | --- | --- | --- | --- | --- | --- | --- | --- | --- | --- | --- | --- | --- |
| *Euglenozoa* | 1.00 | 1.00 | 1.00 | 1.00 | 1.00 | 1.00 | 1.00 | 1.00 | 0.89 | 0.67 | 0.78 | 0.56 | 0.67 | 0.11 | 0.33 | 0.11 | 0.11 | 0.00 | 0.11 | 0.11 | 0.11 | 0.11 | 0.00 | 0.00 | 0.00 |
| *Stramenopiles* | 1.00 | 1.00 | 1.00 | 1.00 | 1.00 | 1.00 | 1.00 | 1.00 | 1.00 | 0.97 | 1.00 | 0.95 | 0.92 | 0.92 | 0.78 | 0.65 | 0.59 | 0.62 | 0.57 | 0.51 | 0.38 | 0.32 | 0.49 | 0.19 | 0.30 |
| *Rhodophyta* | 1.00 | 1.00 | 1.00 | 1.00 | 1.00 | 1.00 | 1.00 | 1.00 | 0.97 | 0.93 | 0.80 | 0.78 | 0.73 | 0.57 | 0.48 | 0.40 | 0.33 | 0.33 | 0.20 | 0.15 | 0.15 | 0.12 | 0.15 | 0.03 | 0.05 |
| *Chlorophyta* | 1.00 | 1.00 | 1.00 | 1.00 | 1.00 | 1.00 | 1.00 | 1.00 | 0.98 | 0.96 | 0.94 | 0.90 | 0.93 | 0.86 | 0.83 | 0.80 | 0.74 | 0.77 | 0.59 | 0.67 | 0.57 | 0.50 | 0.42 | 0.49 | 0.41 |
| *Zygnemophyceae* | 1.00 | 1.00 | 1.00 | 1.00 | 1.00 | 1.00 | 1.00 | 1.00 | 1.00 | 1.00 | 0.82 | 0.91 | 1.00 | 0.82 | 1.00 | 0.64 | 0.55 | 0.73 | 0.91 | 0.45 | 0.55 | 0.64 | 0.55 | 0.27 | 0.09 |
| *Bryophyta* | 1.00 | 1.00 | 1.00 | 1.00 | 1.00 | 1.00 | 1.00 | 1.00 | 1.00 | 1.00 | 1.00 | 1.00 | 1.00 | 0.75 | 1.00 | 0.75 | 0.75 | 0.50 | 0.63 | 0.63 | 0.63 | 0.63 | 0.50 | 0.50 | 0.75 |
| *Polypodiopsida* | 1.00 | 1.00 | 1.00 | 1.00 | 1.00 | 1.00 | 1.00 | 1.00 | 0.98 | 0.94 | 0.86 | 0.73 | 0.73 | 0.39 | 0.47 | 0.45 | 0.37 | 0.10 | 0.18 | 0.16 | 0.10 | 0.12 | 0.14 | 0.14 | 0.12 |
| *Acrogymnospermae* | 1.00 | 1.00 | 1.00 | 1.00 | 1.00 | 1.00 | 1.00 | 1.00 | 1.00 | 0.93 | 0.85 | 0.88 | 0.88 | 0.68 | 0.58 | 0.55 | 0.73 | 0.59 | 0.38 | 0.35 | 0.39 | 0.18 | 0.13 | 0.20 | 0.18 |
| *Basal magnoliophyta* | 1.00 | 1.00 | 1.00 | 1.00 | 1.00 | 1.00 | 1.00 | 1.00 | 0.92 | 0.92 | 0.92 | 0.62 | 0.77 | 0.23 | 0.54 | 0.00 | 0.23 | 0.31 | 0.15 | 0.15 | 0.15 | 0.31 | 0.00 | 0.08 | 0.23 |
| *Magnoliidae* | 1.00 | 1.00 | 1.00 | 1.00 | 1.00 | 1.00 | 1.00 | 1.00 | 1.00 | 0.98 | 1.00 | 0.98 | 0.76 | 0.41 | 0.78 | 0.32 | 0.51 | 0.15 | 0.51 | 0.37 | 0.39 | 0.07 | 0.02 | 0.02 | 0.00 |
| *Alismatales* | 1.00 | 1.00 | 1.00 | 1.00 | 1.00 | 1.00 | 1.00 | 1.00 | 1.00 | 0.93 | 0.93 | 0.93 | 1.00 | 0.50 | 0.79 | 0.71 | 0.57 | 0.36 | 0.64 | 0.57 | 0.36 | 0.21 | 0.21 | 0.21 | 0.14 |
| *Dioscoreales* | 1.00 | 1.00 | 1.00 | 1.00 | 1.00 | 1.00 | 1.00 | 1.00 | 1.00 | 0.90 | 1.00 | 0.60 | 0.70 | 0.50 | 0.60 | 0.50 | 0.60 | 0.30 | 0.40 | 0.50 | 0.10 | 0.00 | 0.30 | 0.20 | 0.00 |
| *Liliales* | 1.00 | 1.00 | 1.00 | 1.00 | 1.00 | 1.00 | 1.00 | 1.00 | 1.00 | 1.00 | 1.00 | 0.98 | 0.80 | 0.46 | 0.37 | 0.73 | 0.46 | 0.12 | 0.66 | 0.49 | 0.24 | 0.39 | 0.15 | 0.07 | 0.51 |
| *Asparagales* | 1.00 | 1.00 | 1.00 | 1.00 | 1.00 | 1.00 | 1.00 | 1.00 | 0.99 | 1.00 | 0.85 | 0.95 | 0.90 | 0.84 | 0.65 | 0.77 | 0.78 | 0.83 | 0.51 | 0.46 | 0.21 | 0.27 | 0.14 | 0.22 | 0.09 |
| *Commelinids* | 1.00 | 1.00 | 1.00 | 1.00 | 1.00 | 1.00 | 1.00 | 0.98 | 1.00 | 1.00 | 1.00 | 0.84 | 0.40 | 0.49 | 0.79 | 0.45 | 0.74 | 0.34 | 0.40 | 0.20 | 0.31 | 0.79 | 0.04 | 0.06 | 0.04 |
| *Early-diverging eudicotyledons* | 1.00 | 1.00 | 1.00 | 1.00 | 1.00 | 1.00 | 1.00 | 1.00 | 1.00 | 0.98 | 0.80 | 0.78 | 0.55 | 0.51 | 0.24 | 0.59 | 0.57 | 0.20 | 0.69 | 0.31 | 0.20 | 0.14 | 0.16 | 0.06 | 0.12 |
| *Santalales* | 1.00 | 1.00 | 1.00 | 1.00 | 1.00 | 1.00 | 1.00 | 1.00 | 0.78 | 0.89 | 0.56 | 0.78 | 0.67 | 0.44 | 0.67 | 0.44 | 0.56 | 0.11 | 0.22 | 0.22 | 0.11 | 0.22 | 0.11 | 0.00 | 0.00 |
| *Saxifragales* | 1.00 | 1.00 | 1.00 | 1.00 | 1.00 | 1.00 | 1.00 | 1.00 | 1.00 | 1.00 | 0.90 | 0.80 | 0.70 | 0.80 | 0.90 | 0.30 | 0.30 | 0.10 | 0.20 | 0.10 | 0.10 | 0.10 | 0.10 | 0.20 | 0.10 |
| *Caryophyllales* | 1.00 | 1.00 | 1.00 | 1.00 | 1.00 | 1.00 | 1.00 | 1.00 | 0.97 | 0.97 | 0.78 | 0.78 | 0.81 | 0.72 | 0.47 | 0.34 | 0.50 | 0.22 | 0.44 | 0.63 | 0.13 | 0.19 | 0.25 | 0.22 | 0.13 |
| *Asterids* | 1.00 | 1.00 | 1.00 | 1.00 | 1.00 | 1.00 | 1.00 | 1.00 | 0.96 | 0.98 | 0.56 | 0.81 | 0.51 | 0.56 | 0.46 | 0.40 | 0.45 | 0.30 | 0.60 | 0.12 | 0.06 | 0.19 | 0.07 | 0.04 | 0.01 |
| *Rosids* | 1.00 | 1.00 | 1.00 | 1.00 | 1.00 | 1.00 | 1.00 | 1.00 | 0.99 | 0.99 | 0.92 | 0.89 | 0.83 | 0.64 | 0.63 | 0.47 | 0.71 | 0.42 | 0.49 | 0.27 | 0.23 | 0.18 | 0.15 | 0.09 | 0.10 |
| Rest | 1.00 | 1.00 | 1.00 | 1.00 | 1.00 | 1.00 | 1.00 | 1.00 | 0.97 | 0.97 | 0.81 | 0.80 | 0.53 | 0.53 | 0.53 | 0.41 | 0.66 | 0.39 | 0.36 | 0.24 | 0.22 | 0.27 | 0.10 | 0.08 | 0.05 |

Supplementary Table S1: Incidence of IRs. This table represents ratio of presence of IRs by its length. Values were calculated by following formula: *amount of sequences containing at least one IR of given length in a subgroup / total amount of sequences in a subgroup.* E.g. in *Alismatales* subgroup there is totally 14 sequences, 9 of those sequences have IRs of length 24, thus 9 / 14 = 0.64.

|  | *Euglenozoa* | *Stramenopiles* | *Rhodophyta* | *Chlorophyta* | *Zygnemophyceae* | *Bryophyta* | *Polypodiopsida* | *Acrogymnospermae* | *Basal magnoliophyta* | *Magnoliidae* | *Alismatales* | *Dioscoreales* |
| --- | --- | --- | --- | --- | --- | --- | --- | --- | --- | --- | --- | --- |
| Amount of sequences | 9 | 37 | 60 | 90 | 11 | 8 | 49 | 85 | 13 | 41 | 14 | 10 |
| Average size of sequence | 95759 | 125870 | 182394 | 162125 | 159540 | 129087 | 149913 | 130661 | 158717 | 155981 | 162219 | 138301 |
| Shortest sequence | 74746 | 89599 | 149987 | 71666 | 129954 | 122630 | 127840 | 107122 | 146859 | 114622 | 143877 | 39386 |
| Longest sequence | 143171 | 165809 | 610063 | 521168 | 207850 | 149016 | 157260 | 166341 | 165389 | 161486 | 169337 | 162477 |
| Length of all | 861832 | 4657181 | 10943643 | 14591263 | 1754937 | 1032697 | 7345751 | 11106178 | 2063317 | 6395213 | 2271071 | 1383009 |
| Q1 size | 85392 | 117514 | 167839.5 | 108796.25 | 137605 | 122893.75 | 150685 | 119739 | 158830 | 152780 | 159496.75 | 152901 |
| Q2 size (median) | 91616 | 122660 | 171284 | 157916.5 | 142017 | 123868.5 | 151126 | 127659 | 159881 | 159443 | 163856 | 154205 |
| Q3 size | 97245 | 130584 | 178391 | 200109.25 | 183356.5 | 130626.75 | 151934 | 134337 | 160866 | 160053 | 165674 | 155917 |
| Avg. frequency | 67.87 | 57.02 | 59.00 | 60.95 | 51.35 | 67.24 | 37.65 | 43.80 | 40.11 | 40.14 | 46.20 | 47.34 |
| Min. frequency | 55.64 | 43.44 | 33.98 | 26.81 | 32.43 | 44.48 | 34.37 | 38.07 | 37.94 | 39.21 | 40.25 | 42.17 |
| Max. frequency | 79.02 | 69.41 | 83.19 | 101.63 | 63.93 | 78.30 | 51.83 | 49.84 | 42.11 | 42.82 | 50.09 | 63.09 |
| Q1 frequency | 64.39 | 54.40 | 54.61 | 49.44 | 48.82 | 65.54 | 35.94 | 40.71 | 39.99 | 39.57 | 45.06 | 42.74 |
| Q2 frequency (median) | 66.19 | 57.20 | 57.75 | 59.28 | 53.96 | 73.13 | 36.49 | 41.45 | 40.04 | 39.90 | 46.55 | 43.88 |
| Q3 frequency | 73.27 | 59.98 | 63.38 | 69.57 | 55.58 | 75.34 | 38.06 | 47.73 | 40.55 | 40.12 | 47.68 | 50.39 |
| Usual longest IR | 18 | 25 | 19 | 27 | 24 | 32 | 18 | 23 | 18 | 18 | 22 | 22 |
| Longest IR | 54 | 60 | 49 | 60 | 48 | 46 | 39 | 44 | 40 | 33 | 60 | 38 |
| Longest IR count | 1 | 7 | 1 | 2 | 1 | 1 | 1 | 1 | 1 | 1 | 2 | 2 |
| Amount of IR | 59456 | 267169 | 657724 | 899336 | 88984 | 68513 | 276037 | 486403 | 82790 | 256651 | 104972 | 63396 |

Supplementary Table S2a: Statistical evaluation of results. This table contains statistical data about groups of sequences. Row denoted *usual longest IR* contains length of inverted repeat that is present roughly in a half of sequences of that group – see S3.

|  | *Liliales* | *Asparagales* | *Commelinids* | *Early-diverging eudicotyledons* | *Santalales* | *Saxifragales* | *Caryophyllales* | *Asterids* | *Rosids* | *Rest* |
| --- | --- | --- | --- | --- | --- | --- | --- | --- | --- | --- |
| Amount of sequences | 41 | 125 | 290 | 49 | 9 | 10 | 32 | 398 | 522 | 662 |
| Average size of sequence | 153915 | 154179 | 139103 | 158422 | 131202 | 153541 | 151065 | 152204 | 156817 | 146372 |
| Shortest sequence | 150576 | 142996 | 113490 | 147378 | 118743 | 147048 | 113064 | 35336 | 19400 | 11348 |
| Longest sequence | 158229 | 178131 | 181397 | 166758 | 156154 | 160410 | 192912 | 176331 | 242575 | 269857 |
| Length of all | 6310502 | 19272364 | 40339734 | 7762654 | 1180820 | 1535414 | 4834081 | 60577170 | 81858350 | 96897958 |
| Q1 size | 152145 | 151788 | 136055.75 | 155832 | 122562 | 151006.5 | 150444.5 | 151912.5 | 154599.5 | 136794.5 |
| Q2 size (median) | 152677 | 153953 | 139171.5 | 157817 | 128744 | 152692.5 | 151686 | 153377 | 159441.5 | 155196 |
| Q3 size | 156746 | 157043 | 140119.5 | 160357 | 131016 | 156115.75 | 153450.75 | 156813.75 | 160886 | 160126 |
| Avg. frequency | 45.13 | 44.01 | 40.63 | 41.54 | 44.56 | 41.44 | 44.89 | 42.80 | 45.28 | 46.45 |
| Min. frequency | 42.48 | 41.56 | 38.26 | 39.14 | 40.86 | 40.24 | 40.12 | 38.06 | 35.12 | 28.33 |
| Max. frequency | 46.16 | 63.66 | 51.64 | 44.81 | 47.82 | 43.44 | 47.04 | 60.80 | 74.79 | 191.98 |
| Q1 frequency | 44.30 | 42.85 | 40.15 | 40.71 | 42.28 | 40.51 | 43.53 | 42.11 | 44.57 | 40.17 |
| Q2 frequency (median) | 45.71 | 43.31 | 40.51 | 41.61 | 43.96 | 41.49 | 45.28 | 42.73 | 45.44 | 42.51 |
| Q3 frequency | 45.92 | 43.89 | 40.83 | 42.09 | 46.79 | 42.03 | 46.22 | 43.44 | 46.27 | 45.15 |
| Usual longest IR | 18 | 24 | 17 | 19 | 18 | 20 | 19 | 19 | 20 | 20 |
| Longest IR | 36 | 60 | 37 | 40 | 28 | 30 | 35 | 60 | 60 | 60 |
| Longest IR count | 1 | 3 | 1 | 2 | 1 | 1 | 1 | 2 | 1 | 9 |
| Amount of IR | 284686 | 848495 | 1640815 | 322481 | 52549 | 63628 | 217065 | 2583537 | 3696845 | 4305421 |

Supplementary Table S2b: Statistical evaluation of results. This table contains statistical data about groups of sequences. Row denoted *usual longest IR* contains length of inverted repeat that is present roughly in a half of sequences of that group – see S3.

|  | *Feature count* | *Feature total length* | *Avg. feature size* |
| --- | --- | --- | --- |
| gene | 343857 | 268130351 | 780 |
| CDS | 226783 | 177415046 | 782 |
| misc_feature | 7492 | 67388213 | 8995 |
| repeat_region | 3584 | 48285000 | 13472 |
| rRNA | 18719 | 21740714 | 1161 |
| exon | 36325 | 10989197 | 303 |
| intron | 11028 | 8864648 | 804 |
| tRNA | 91586 | 6362382 | 69 |
| stem_loop | 241 | 13527 | 56 |
| regulátory | 371 | 1987 | 5 |

Supplementary Table S3: Feature amounts and length. This table shows amounts of annotated features in all downloaded sequences and their length.

|  | *all inside* | *8+ inside* | *10+ inside* | *12+ inside* | *all around* | *8+ around* | *10+ around* | *12+ around* | *all before* | *8+ before* | *10+ before* | *12+ before* | *all after* | *8+ after* | *10+ after* | *12+ after* |
| --- | --- | --- | --- | --- | --- | --- | --- | --- | --- | --- | --- | --- | --- | --- | --- | --- |
| gene | 39.4991 | 5.4413 | 0.7550 | 0.1557 | 53.7465 | 10.1879 | 2.2400 | 0.7071 | 49.1549 | 9.0059 | 1.7788 | 0.4089 | 58.3382 | 11.3698 | 2.7012 | 1.0053 |
| CDS | 39.4546 | 5.3510 | 0.6799 | 0.1270 | 56.8928 | 10.5302 | 2.2743 | 0.7032 | 52.1417 | 9.3112 | 1.7271 | 0.3775 | 61.6438 | 11.7492 | 2.8215 | 1.0289 |
| misc_feature | 46.8330 | 7.9121 | 1.5010 | 0.4243 | 40.7461 | 6.2493 | 1.0805 | 0.2910 | 34.9333 | 4.8091 | 0.7461 | 0.2122 | 46.5590 | 7.6895 | 1.4148 | 0.3697 |
| repeat_region | 38.8865 | 5.6452 | 0.9641 | 0.2645 | 50.2525 | 13.1808 | 5.7520 | 3.2840 | 49.3945 | 12.1763 | 4.9051 | 3.0859 | 51.1105 | 14.1853 | 6.5988 | 3.4821 |
| rRNA | 28.6355 | 2.5199 | 0.2380 | 0.0150 | 36.3794 | 4.2940 | 0.7346 | 0.2399 | 33.3367 | 2.6743 | 0.3649 | 0.1234 | 39.4220 | 5.9138 | 1.1042 | 0.3563 |
| exon | 34.5543 | 4.0200 | 0.3726 | 0.0435 | 45.6087 | 7.6851 | 1.2852 | 0.2198 | 38.8182 | 5.7299 | 0.9586 | 0.1399 | 52.3992 | 9.6402 | 1.6118 | 0.2998 |
| intron | 47.3651 | 8.3139 | 1.5275 | 0.3732 | 38.9413 | 4.9973 | 0.7241 | 0.0875 | 37.5617 | 4.3444 | 0.8841 | 0.0680 | 40.3210 | 5.6502 | 0.5640 | 0.1070 |
| tRNA | 35.9296 | 3.7329 | 0.4558 | 0.2650 | 47.9495 | 9.9725 | 2.4264 | 0.7127 | 45.4892 | 9.5946 | 2.2378 | 0.5419 | 50.4098 | 10.3505 | 2.6149 | 0.8834 |
| stem_loop | 65.9422 | 14.8592 | 4.1399 | 1.9960 | 69.6681 | 16.8880 | 6.6183 | 4.2116 | 64.2739 | 13.8174 | 4.1909 | 1.8257 | 75.0622 | 19.9585 | 9.0456 | 6.5975 |
| regulatory | 1.5098 | 0.5033 | 0.5033 | 0.5033 | 75.8760 | 14.5148 | 3.2210 | 0.7412 | 84.5822 | 17.6011 | 3.8814 | 0.9164 | 67.1698 | 11.4286 | 2.5607 | 0.5660 |

Supplementary Table S4: Frequency of IRs per 1000 bp around annotated features. This table presents frequency of IRs around, before and after annotated features and frequency of IRs which overlap with annotated features (inside). The length of sub-sequence for IRs presence around, before and after was set to 100 bp. It means that if inverted repeat of length e.g. 10 was found 70 bases before annotated feature start, it was considered present in the surrounding of that feature. See Figure S3 for graphical explanation of classification.

|  | *all inside* | *8+ inside* | *10+ inside* | *12+ inside* | *all around* | *8+ around* | *10+ around* | *12+ around* | *all before* | *8+ before* | *10+ before* | *12+ before* | *all after* | *8+ after* | *10+ after* | *12+ after* |
| --- | --- | --- | --- | --- | --- | --- | --- | --- | --- | --- | --- | --- | --- | --- | --- | --- |
| CDS | 0.9989 | 0.9834 | 0.9005 | 0.8156 | 1.0585 | 1.0336 | 1.0153 | 0.9945 | 1.0608 | 1.0339 | 0.9709 | 0.9232 | 1.0567 | 1.0334 | 1.0445 | 1.0234 |
| misc_feature | 1.1857 | 1.4541 | 1.9881 | 2.7244 | 0.7581 | 0.6134 | 0.4824 | 0.4115 | 0.7107 | 0.5340 | 0.4195 | 0.5191 | 0.7981 | 0.6763 | 0.5238 | 0.3678 |
| repeat_region | 0.9845 | 1.0375 | 1.2769 | 1.6983 | 0.9350 | 1.2938 | 2.5678 | 4.6445 | 1.0049 | 1.3520 | 2.7576 | 7.5477 | 0.8761 | 1.2476 | 2.4429 | 3.4638 |
| rRNA | 0.7250 | 0.4631 | 0.3153 | 0.0963 | 0.6769 | 0.4215 | 0.3279 | 0.3392 | 0.6782 | 0.2969 | 0.2051 | 0.3018 | 0.6757 | 0.5201 | 0.4088 | 0.3544 |
| exon | 0.8748 | 0.7388 | 0.4935 | 0.2793 | 0.8486 | 0.7543 | 0.5738 | 0.3109 | 0.7897 | 0.6362 | 0.5389 | 0.3420 | 0.8982 | 0.8479 | 0.5967 | 0.2982 |
| intron | 1.1991 | 1.5279 | 2.0232 | 2.3961 | 0.7245 | 0.4905 | 0.3232 | 0.1237 | 0.7641 | 0.4824 | 0.4970 | 0.1663 | 0.6912 | 0.4969 | 0.2088 | 0.1064 |
| tRNA | 0.9096 | 0.6860 | 0.6037 | 1.7016 | 0.8921 | 0.9789 | 1.0832 | 1.0079 | 0.9254 | 1.0654 | 1.2580 | 1.3254 | 0.8641 | 0.9103 | 0.9681 | 0.8788 |
| stem_loop | 1.6695 | 2.7308 | 5.4831 | 12.8163 | 1.2962 | 1.6577 | 2.9546 | 5.9564 | 1.3076 | 1.5343 | 2.3560 | 4.4654 | 1.2867 | 1.7554 | 3.3487 | 6.5627 |
| regulatory | 0.0382 | 0.0925 | 0.6666 | 3.2315 | 1.4117 | 1.4247 | 1.4380 | 1.0483 | 1.7207 | 1.9544 | 2.1820 | 2.2415 | 1.1514 | 1.0052 | 0.9480 | 0.5631 |

Supplementary Table S5: IRs ratio around features to Gene feature. This table shows ratio of IRs frequency around features to frequency of IRs around *gene* feature. The *rRNA* feature has frequency of overlapping (inside) IRs of length 8 and more of value 2.5199 and *gene* feature has frequency 5.4413, therefore the ratio is calculated as 2.5199 / 5.4413 = 0.4631. It means that *rRNA* feature has about a half less overlapping IRs as *gene* feature.
