# Supplementary figures and images for: Complex Analyses of Short Inverted Repeats in All Sequenced Chloroplast DNAs
Source: Biomed Res Int. 2018 Jul 24;2018:1097018. doi: 10.1155/2018/1097018 (PMC6081594; doi:10.1155/2018/1097018)

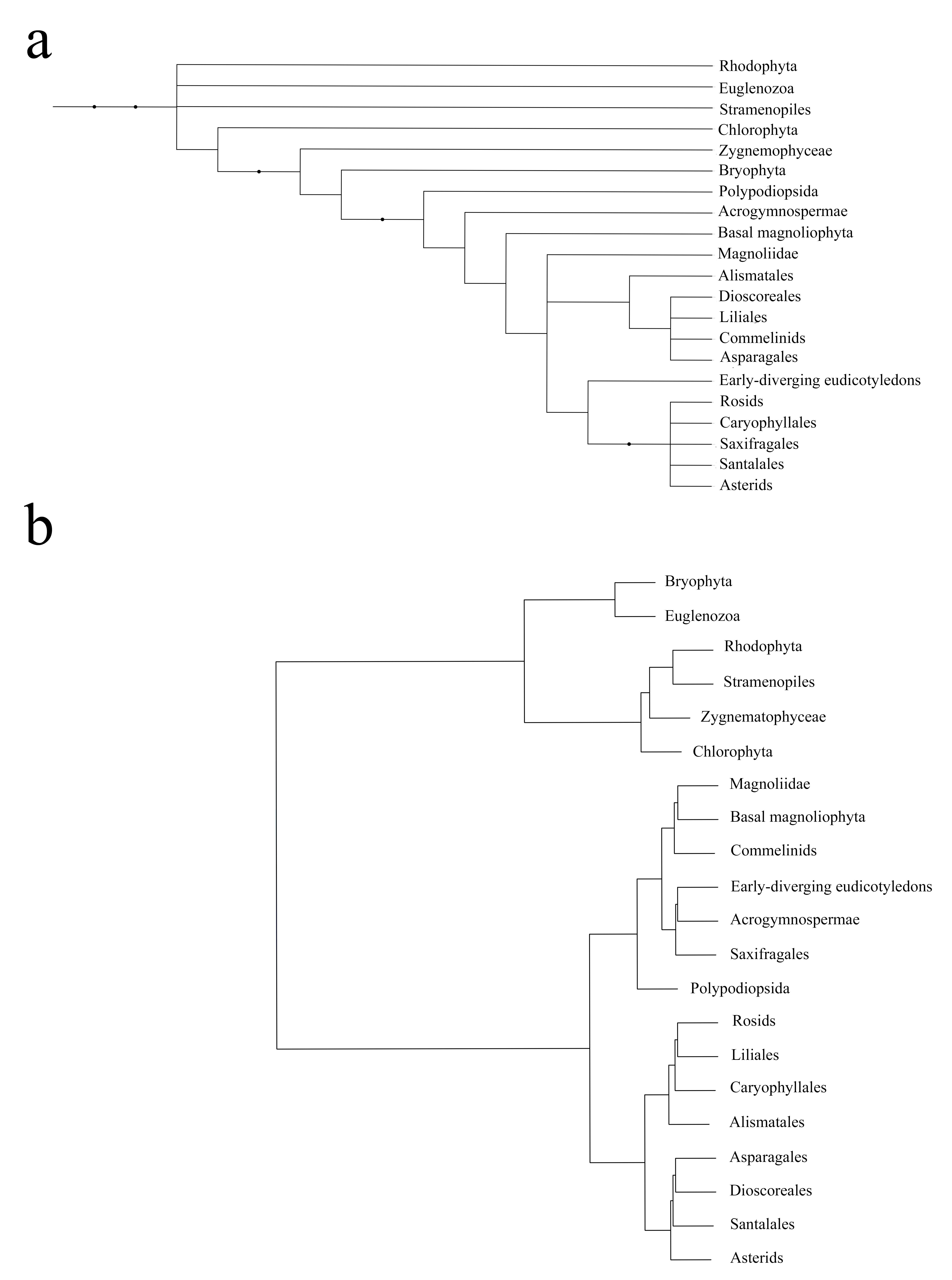

Supplement: Supplementary Material — Supplementary Figure S1: neighborhood of an annotated feature. Example of possible S-IR occurrence around features and its classification: (a) an S-IR overlapping only partially with a feature is considered to be in near neighborhood; (b) an S-IR overlapping fully with a feature is therefore considered to be inside; (c) an S-IR is not considered to be in near neighborhood because it is not fully overlapping with either a feature or its neighborhood. Supplementary Figure 2: phylogenetic tree of all inspected organisms with chloroplast genome made using iTOL. Subgroups are highlighted by different colors. From left counterclockwise: Rosids (red, 522 species); Asterids (blue, 398 species); Caryophyllales (dark green, 32 species); Saxifragales (yellow, 10 species); Santalales (purple, 9 species); Early-Diverging Eudicotyledons (green, 49 species); Commelinids (red, 290 species); Asparagales (blue, 125 species); Liliales (yellow, 41 species); Dioscoreales (purple, 10 species); Alismatales (dark green, 14 species); Magnoliidae (orange, 41 species); Basal Magnoliophyta (green, 13 species); Acrogymnospermae (red, 85 species); Polypodiopsida (green, 49); Bryophyta (orange, 8 species); Zygnemophyceae (red, 11 species); Chlorophyta (purple, 90 species); Rhodophyta (green, 60 species); Stramenopiles (orange, 37 species); Euglenozoa (blue, 9 species). Supplementary Code S1: method for construction of interactive PCA plots from S-IR data by R (version 3.4.0). Referred Excel input for this analysis was values from even S-IR length columns of Supplementary Table S1. Supplementary Table S1: incidence of S-IRs. This table represents ratio of presence of S-IRs by their length. Values were calculated by the following formula: number of sequences containing at least one S-IR of given length in a subgroup/total number of sequences in a subgroup. For example, in Alismatales subgroup, there is a total of 14 S-IR sequences, 9 of those sequences have S-IRs of length 24, and thus 9 / 14 = 0.6 [file 1097018.f1.zip › Supplementary Fig1.tif]

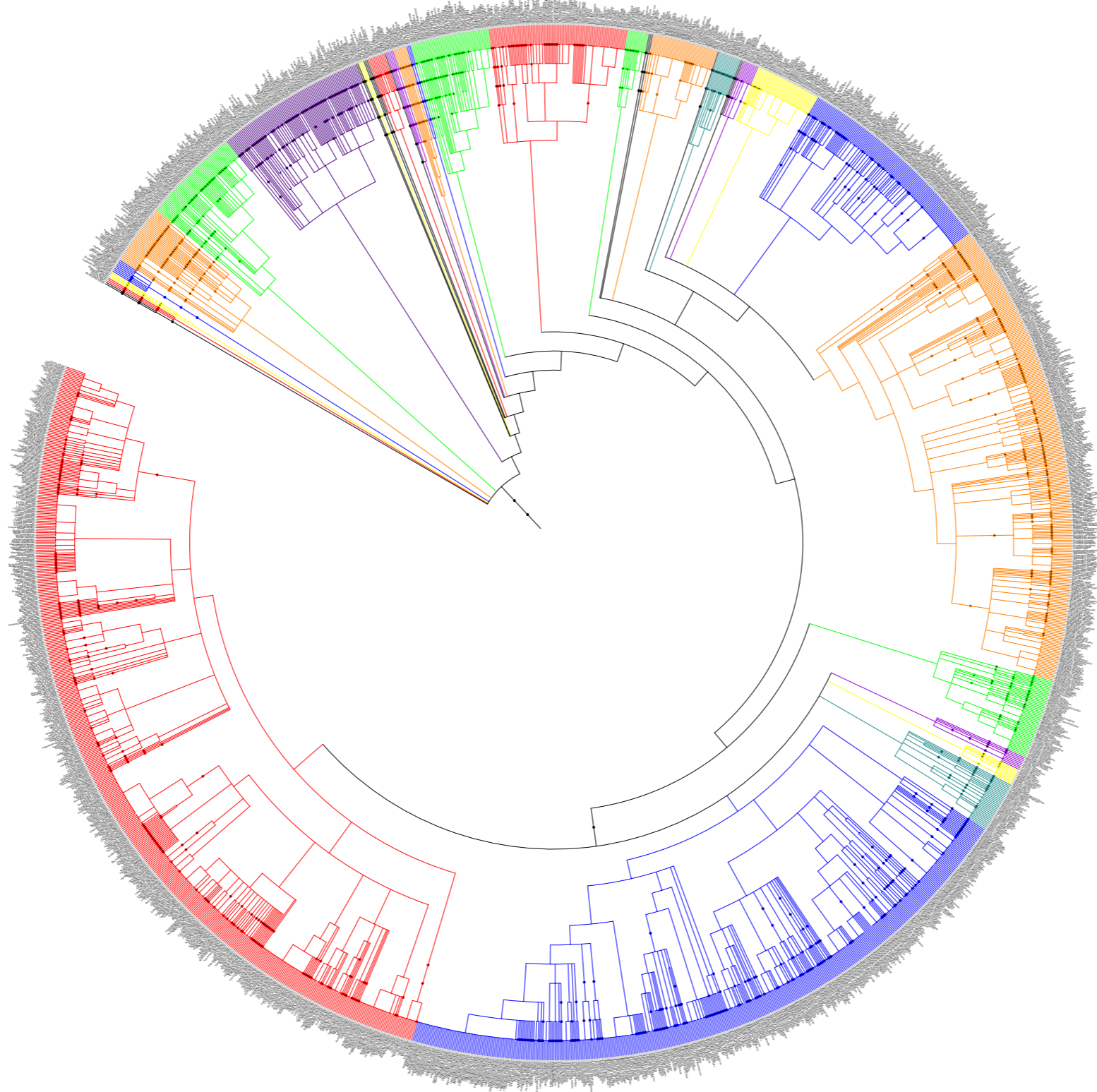

Supplement: Supplementary Material — Supplementary Figure S1: neighborhood of an annotated feature. Example of possible S-IR occurrence around features and its classification: (a) an S-IR overlapping only partially with a feature is considered to be in near neighborhood; (b) an S-IR overlapping fully with a feature is therefore considered to be inside; (c) an S-IR is not considered to be in near neighborhood because it is not fully overlapping with either a feature or its neighborhood. Supplementary Figure 2: phylogenetic tree of all inspected organisms with chloroplast genome made using iTOL. Subgroups are highlighted by different colors. From left counterclockwise: Rosids (red, 522 species); Asterids (blue, 398 species); Caryophyllales (dark green, 32 species); Saxifragales (yellow, 10 species); Santalales (purple, 9 species); Early-Diverging Eudicotyledons (green, 49 species); Commelinids (red, 290 species); Asparagales (blue, 125 species); Liliales (yellow, 41 species); Dioscoreales (purple, 10 species); Alismatales (dark green, 14 species); Magnoliidae (orange, 41 species); Basal Magnoliophyta (green, 13 species); Acrogymnospermae (red, 85 species); Polypodiopsida (green, 49); Bryophyta (orange, 8 species); Zygnemophyceae (red, 11 species); Chlorophyta (purple, 90 species); Rhodophyta (green, 60 species); Stramenopiles (orange, 37 species); Euglenozoa (blue, 9 species). Supplementary Code S1: method for construction of interactive PCA plots from S-IR data by R (version 3.4.0). Referred Excel input for this analysis was values from even S-IR length columns of Supplementary Table S1. Supplementary Table S1: incidence of S-IRs. This table represents ratio of presence of S-IRs by their length. Values were calculated by the following formula: number of sequences containing at least one S-IR of given length in a subgroup/total number of sequences in a subgroup. For example, in Alismatales subgroup, there is a total of 14 S-IR sequences, 9 of those sequences have S-IRs of length 24, and thus 9 / 14 = 0.6 [file 1097018.f1.zip › Supplementary Fig2.pdf]
